# Health disparities persist for adults with developmental disabilities: NHIS insights, 1999-2018

**DOI:** 10.1093/haschl/qxae158

**Published:** 2025-04-22

**Authors:** Kiley J McLean, Jamie Koenig, Samara Wolpe, Wei Song, Lauren Bishop

**Affiliations:** A.J. Drexel Autism Institute, Drexel University, 3020 Market Street, Suite 560, Philadelphia, PA 19104-3734, United States; Sandra Rosenbaum School of Social Work, University of Wisconsin-Madison, Madison, WI 53706, United States; Waisman Center, University of Wisconsin-Madison, Madison, WI 53705, United States; Department of Education, University of California, Los Angeles, Los Angeles, CA 90095, United States; A.J. Drexel Autism Institute, Drexel University, 3020 Market Street, Suite 560, Philadelphia, PA 19104-3734, United States; Sandra Rosenbaum School of Social Work, University of Wisconsin-Madison, Madison, WI 53706, United States; Waisman Center, University of Wisconsin-Madison, Madison, WI 53705, United States

**Keywords:** public health, systems of care, disability, adulthood disability, health policy

## Abstract

This study assesses changes in self-reported health and healthcare status among adults with intellectual and developmental disabilities (I/DD) over the past 20 years, utilizing data from the National Health Interview Survey. We conducted a cross-sectional analysis of 601 464 adults 18 and older, categorized by disability status: no functional limitations, developmental disabilities, intellectual disabilities, and other functional limitations. We aimed to identify trends in health status, healthcare access, affordability, and utilization from 1999 to 2018, comparing outcomes across disability groups. Results indicate adults with intellectual disabilities reported poorer health compared to those without functional limitations, particularly in the most recent period (2014-2018). Adults with developmental disabilities exhibited increased odds of poor health during 2009-2013 compared to 1999-2003, but no significant differences occurred between other periods. Additionally, poverty rates for adults with I/DD were higher, with a substantial proportion of adults with intellectual disabilities living in poverty compared to those without functional limitations, indicating persistent disparities without significant trend improvements. Despite advancements in healthcare access and affordability, self-reported health outcomes for adults with I/DD have not improved, highlighting the need for policies to enhance care quality. Future research should focus on effective healthcare practices and provider training to address these persistent disparities.

## Introduction

Intellectual and developmental disabilities (I/DD) often present at birth, affecting physical, learning, language, or behavioral development throughout life.^1^ Recent studies indicate that about 17% of children have one or more I/DD, a notable increase over the last 20 years, primarily due to rises in attention-deficit/hyperactivity disorder (ADHD), autism, and intellectual disabilities.^1,2^ Adults with I/DD face increased mortality risk and high rates of chronic conditions such as obesity, asthma, diabetes, cardiovascular disease, and chronic pain, as well as higher rates of epilepsy and psychiatric disorders compared to those without I/DD.^3-7^

The umbrella term “intellectual and developmental disability” encompasses a broad spectrum of diagnoses and varying degrees of functional limitations. Conditions such as cerebral palsy (which can range from mild to severe), Down syndrome, ADHD, and autism can lead to diverse challenges that impact health outcomes. Some adults experience structural and physical health challenges that continue to affect their self-reported health, even with optimal healthcare access.^8^ Adverse health outcomes in I/DD populations have been linked to personal factors like physical inactivity and poor nutrition. However, recent literature suggests these outcomes result from health disparities, including inequitable access to high-quality medical care, inadequate healthcare provider training, and exclusion from public health efforts.^9-12^ Individuals with I/DD in the United States show limited use of health services and less frequent primary healthcare visits, even with coverage.^13-15^

A study using electronic health records in Ohio found that individuals with I/DD were significantly less likely to visit specialized and preventative care providers, such as gastroenterologists, pulmonologists, cardiologists, obstetrician gynecologists (OB/GYNs), general surgeons, and pain management specialists.^16^ This lack of access to preventative and inclusive healthcare is a significant problem, given the high rates of co-occurring conditions among people with I/DD.^11,17,18^

The current literature indicates mixed findings regarding the impact of the Affordable Care Act (ACA) on the health and longevity of individuals with disabilities and mental health conditions. Various studies utilizing the similar data sources examine the potential effects of the ACA on healthcare access, chronic illness management, and overall health outcomes, revealing inconsistencies and nuanced outcomes that warrant further exploration. Notably, Kaye^19^ presents a detailed analysis of how the ACA has affected different disability groups, emphasizing that while some individuals have experienced improved access to care, others continue to face significant barriers, particularly concerning specialized services. There are persistent challenges in access to healthcare for individuals with disabilities, with factors such as socioeconomic status, geographic location, and systemic biases contributing to ongoing disparities, before and after the full implementation of the ACA.^20,21^ Given these complexities and the ongoing challenges faced by individuals with I/DD, it is crucial to investigate how recent policy initiatives have influenced their health outcomes.

### Purpose

Over the past 20 years, the United States has implemented several policy initiatives to reduce health disparities, including the Patient Protection and ACA of 2010.^22^ The ACA aimed to improve healthcare access by expanding public health coverage and making private insurance more affordable, particularly for those with preexisting conditions like I/DD.^19^ While emerging research has begun to assess the impact of these policies, much remains unknown about their effect on individuals with I/DD.^23^

This study uses National Health Interview Survey (NHIS) data to examine 20-year trends in health status and healthcare access, affordability, and utilization by disability status. We aim to determine if the health and healthcare status of adults with I/DD has changed, assess the impact of policies like the ACA, and identify targets for reducing health disparities in future healthcare policy reforms. Specifically, our research questions are:

1. How has the health status of adults with I/DD in the United States changed over the past 20 years?
2. What are the trends in healthcare access, utilization, and affordability for adults with I/DD in the United States?

We hypothesize that policy changes will reveal increased healthcare affordability and accessibility, alongside improved health status. This is the first study to evaluate national, long-term trends among non-institutionalized adults with I/DD in the United States.

## Methods

### Data source

Data were obtained from the NHIS, a cross-sectional household interview survey of the civilian non-institutionalized US population, accessed via IPUMS USA.^24-26^ The NHIS uses a complex, multi-stage probability sample to allow for nationally representative estimates.^27,28^ This study was exempt from IRB review at University of Wisconsin-Madison.

### Sample

Adults aged 18 and older from the NHIS sample adult file (1999-2018) were included. Information about the adult sampled from each household (sample adult) is self-reported unless the individual is physically or mentally unable to self-report, in which case a knowledgeable proxy can answer for the sample adult. This approach ensures that health outcomes and experiences accurately reflect individuals’ perceptions while allowing for valid data collection when direct self-reporting is not feasible. We excluded those with missing disability status data, resulting in a final sample of 601 464 adults (1676 observations deleted). “Sample adults” included 384 586 adults with no functional limitation, 580 with developmental disabilities, 814 with intellectual disabilities, and 215 484 with other functional limitations. The decision to analyze these groups separately was made to better understand the unique health outcomes and challenges faced by each population. Each group may experience different health issues and barriers that warrant individualized examination.^23^ Missing rates for key variables were generally low. Except for poverty status (14%), missing rates among demographic variables were <1%. Similarly, except for healthcare access (15%), missing rates of healthcare outcomes were <1.5%.

### Demographic variables

We identified sample adults with I/DD in the NHIS by including adults with “any functional limitation;” AND “functional limitation from: intellectual disability” OR “functional limitation from: other developmental problem.” Functional limitation was based on a recoded variable indicating whether adults had difficulty doing any of several specific activities because of a health problem. “Health problem” was defined by interviewers “as any physical, mental, or emotional problem or illness.” If a person acknowledged having any difficulty with activities such as: walking a quarter of a mile or 10 steps without resting, participating in social activities, relaxing at home or doing things for leisure, or pushing or pulling large objects, they were classified as “limited in any way.” If an adult was limited in any way as defined above, they were asked follow-up questions about what condition or health problem was causing these difficulties. Having a “developmental problem, such as cerebral palsy” was one of the possible conditions on the interviewer-provided flashcard. Intellectual disability (termed “Mental Retardation” during some waves of the NHIS), was also one of the possible conditions. Based on this, we created a disability status variable indicating if each sample adult had (1) no functional limitations, (2) developmental disabilities, (3) intellectual disabilities, or (4) any other type of functional limitation.

Demographic characteristics that are likely related to healthcare utilization and health status were also extracted (race, ethnicity, sex, age, poverty status, educational attainment, employment, region of residence). *Race and ethnicity* were self-reported: (1) Non-Hispanic White, (2) Non-Hispanic Black/African American, (3) Non-Hispanic Asian or Pacific Islander, (4) Hispanic/Latino, or (5) Non-Hispanic other. *Sex* indicated: (1) male or (2) female. *Poverty status* indicated: family income (1) at or above or (2) below the poverty level. The NHIS determines if a family or individual is at or above the poverty line by comparing their income to the federal poverty threshold. The federal poverty threshold is adjusted annually for inflation by the US Census Bureau and varies by family size and composition.^27^  *Educational attainment* reported highest level of schooling completed: (1) less than high school, (2) high school diploma or General Educational Development Test (GED), (3) some college, or (4) bachelor's degree or higher. *Employment status* reported whether adults were: (1) working in the past week including working for pay, performing seasonal or contract work, or working at a job or business NOT for pay; OR whether adults were: (2) part of the labor force but unemployed, or (3) out of the labor force entirely. *Region of residence* indicated: region of housing unit containing the survey participant. The 4 regions—(1) Northeast, (2) North Central, Midwest, (3) South, and (4) West—correspond to regions recognized by the US Census Bureau.

### Study outcomes

The study outcomes are defined below. Similar to past NHIS trend analyses, we set “don’t know,” “refused,” or values without to missing for each outcome.^29^

#### Health Status

Health status was assessed through the NHIS question: “Would you say your health in general is excellent, very good, good, fair, or poor?” Responses were dichotomized into poor/fair health (yes/no).

#### Healthcare access

*Healthcare access* was assessed by whether adults had health insurance and a usual source of care.^29^ This variable was constructed based on several NHIS questions indicating if the sample adult had health insurance and if so, which type and if they had a usual place for medical care and if so, which type of place. Adults were then classified as uninsured if at the time of their interview, they reported not having private insurance, Medicare, Medicaid, military plan, or other government- or state-sponsored health plan. NHIS considered sample adults with only Indian Health Service coverage to be uninsured as well. To conserve power, we recoded sample adults with military health insurance, government-, or state-sponsored health plans as “other health insurance.” Sample adults with a usual source of care were those who had a place they usually go, such as a clinic or doctor's office, when they were sick or needed advice about their health. If an adult indicated that their usual place of care was the emergency room, they were considered to not have a usual source of care.

#### Healthcare affordability

Affordability was assessed by whether adults delayed seeking medical care due to cost, needed but did not get medical care because they could not afford it, and/or needed but did not get prescription medicines due to cost.

#### Healthcare utilization

Utilization was determined by whether the adult had seen or talked to a health professional in the past 12 months. If they had not, they were coded as not having utilized healthcare in the past year.

### Analytic plan

Analyses incorporated strata and weights to produce nationally representative estimates, adjusting sampling weights to represent the US population across the 20-year study period.^30^ Missing data on family income were imputed to determine the poverty threshold indicator.^27^

We summarized demographic characteristics and compared them by disability status using χ^2^ tests. Multivariate logistic regression predicted health and healthcare outcomes across 4 periods (1999-2003, 2004-2008, 2009-2013, and 2014-2018), adjusting for key covariates. Changes in outcomes over time by disability status were examined through weighted proportions and logistic regression models. Observations with missing outcome data were excluded from the model (ie, complete case analysis).

Our post-hoc analysis assessed variations in outcomes by disability status. Given the number of statistical comparisons in this study, however, we employed methods to correct for the family wise error rate (FWER) to reduce type I error.^31^ As the number of hypotheses increases, so does the risk of type I error. While the Bonferroni method is commonly used to manage FWER by dividing the significance level by the number of hypotheses, it can overly reduce power and increase type II error. Therefore, we initially set a *P*-value threshold of .001 to control for FWER and then applied the Holm method, a less conservative stepwise approach that ranks *P*-values and adjusts the alpha level. In this method, we ranked the *P*-values and set the alpha level to .05, leading to a first non-rejected hypothesis at *P* = .151. Consequently, we established a secondary significance level at *P* < .151 for subsequent hypotheses.

## Results

### Demographic comparisons

Table 1 highlights the sociodemographic characteristics of the study population, with a mean age of 46.16 years. The sample included 63.9% with no functional limitation, 0.1% with developmental disabilities, 0.14% with intellectual disabilities, and 35.8% with other functional limitations.

**Table 1.** Sociodemographic characteristics of sample adults, NHIS 1999-2018.

	Full sample	No functional limitation	Developmental disability	Intellectual disability	Other functional limitation	*P*-value^a^
	*N* = 601 464	*N* = 384 586	*N* = 580	*N* = 814	*N* = 215 484	
	Weighted % (95% CI)	Weighted % (95% CI)	Weighted % (95% CI)	Weighted % (95% CI)	Weighted % (95% CI)	
Age (mean)	46.16 (46.03-46.3)	41.35 (41.2-42.5)	39.28 (37-40)	39.28 (37-40)	55.78 (55.6-55.9)	
Race						<.001^a^
Non-Hispanic White	68.97% (68.5-69.4)	66.04% (65.6-66.5)	71.20% (66.3-75.7)	60.44% (55.7-65.0)	74.83%(74.3-75.3)	
Non-Hispanic Black/ African American	11.67% (11.4-11.9)	11.83% (11.5-12.1)	14.24% (11.1-18.1)	21.32% (17.6-25.6)	11.31% (10.9-11.6)	
Non-Hispanic Asian or Pacific Islander	4.64% (4.5-4.8)	5.58% (5.4-5.8)	1.30% (0.7-2.4)	2.21% (1.3-3.8)	2.79% (2.7-2.9)	
Hispanic/Latino	13.68% (13.3-14.0)	15.60% (15.2-15.9)	11.69% (8.6-15.7)	14.74% (11.5-18.7)	9.87% (9.5-10.2)	
Non-Hispanic Other	1.04% (0.9-1.1)	0.96% (0.9-1.0)	1.57% (0.8-3.3)	1.29% (0.6-2.7)	1.20% (1.1-1.3)	
Sex						<.001^a^
Female	51.82% (51.7-52)	48.30% (48.1-48.5)	49.33% (44.1-54.6)	45.19% (40.6-49.8)	58.88% (58.6-59.2)	
Male	48.18% (48-48.4)	51.70% (51.5-51.9)	50.67% (45.5-55.9)	54.81% (60.2-59.4)	41.12% (40.8-41.4)	
Educational attainment						<.001^a^
Less than high school	14.90% (14.7-15.1)	12.70% (12.5-12.9)	26.24% (21.7-31.4)	46.39% (41.6-51.3)	19.10% (18.8-19.4)	
High school diploma or GED	27.47% (27.2-27.7)	25.86% (25.6-26.1)	40.49% (35.5-45.7)	43.59% (38.8-48.5)	30.57% (30.3-30.9)	
Some college	29.94% (29.7-30.2)	30.26% (29.9-30.5)	20.95% (17-25.5)	7.75% (5.5-10.8)	29.43% (29.1-29.7)	
Bachelor's degree or higher	27.69% (27.3-28.1)	31.18% (30.8-31.6)	12.32% (9.6-15.7)	2.28% (1.3-4)	20.90% (20.5-21.3)	
Employment status						<.001^a^
Employed	63.04% (62.8-63.3)	73.57% (73.3-73.8)	27.0% (22.8-31.7)	18.74% (15.3-22.8)	42.43% (42-42.8)	
Unemployed	4.03% (3.9-4.1)	4.24% (4.1-4.3)	5.09% (3.2-7.9)	2.22% (1.3-3.9)	3.63% (3.5-3.7)	
Not in the labor force	32.93% (32.7-33.2)	22.19% (21.9-22.4)	67.91% (63-72.4)	79.04% (74.9-82.7)	53.95% (53.5-54.4)	
Poverty status						<.001^a^
At or above poverty threshold	75.89% (75.6-76.2)	76.90% (76.6-77.2)	69.36% (64.8-73.6)	62.16% (57.6-66.6)	73.98% (73.6-74.3)	
Below poverty threshold	10.48% (13.4-13.9)	9.21% (8.9-9.5)	20.44% (16.9-24.4)	25.32% (21.6-29.5)	12.89% (12.6-13.2)	
Region						<.001^a^
Northeast	18.12% (17.7-18.5)	18.48% (18.1-18.9)	15.78% (12.4-19.9)	17.23% (13.9-21.1)	17.41% (16.9-17.9)	
North Central/Midwest	23.52% (23.1-23.9)	22.60% (22.1-23.1)	25.69% (21.6-30.3)	23.69% (19.9-27.9)	25.34% (24.8-25.9)	
South	36.43% (35.9-36.9)	36.47% (35.9-37.1)	39.42% (34.3-44.8)	39.59% (35.1-44.3)	36.31% (35.7-36.9)	
West	21.93% (21.5-22.4)	22.45% (21.9-22.9)	19.11% (15.4-23.5)	19.49% (16.1-23.4)	20.93% (20.4-21.5)	
Healthcare status						<.001^a^
No insurance	14.97% (14.7-15.2)	16.96% (16.7-17.2)	7.23% (4.9-10.5)	4.78% (3.1-7.4)	11.08% (10.9-11.3)	
Private Insurance	56.45% (56.1-56.8)	65.40% (65.1-65.7)	25.94% (21.8-30.6)	9.48% (7.2-12.5)	39.0% (38.6-39.4)	
Medicaid	6.31% (6.2-6.5)	5.45% (5.3-5.6)	29.63% (24.9-34.8)	46.0% (41.5-50.6)	7.78% (7.6-7.9)	
Medicare	17.59% (17.4-17.8)	8.60% (8.5-8.8)	32.66% (28.3-37.4)	34.94% (30.7-39.4)	35.33% (34.9-35.7)	
Other health insurance	4.57% (4.5-4.8)	3.59% (3.5-3.7)	4.53% (2.5-7.9)	4.80% (3.3-7.0)	6.81% (6.6-7.0)	

Frequencies and percentages are reported for categorical variables. Means and standard deviations are reported for continuous variables. To determine poverty status, the reported total family income for an individual was compared to the US Census Bureau's poverty thresholds for the year in question. These thresholds are based not only on total income but also on family size and the number of children under age 18 in the home. Source: Author's analysis of data from the National Health Interview Survey, 1999-2018.

Abbreviation: NHIS, National Health Interview Survey.

^a^ *P*-value from χ^2^ test of independence.

Key differences emerged between groups in race, education, employment, and poverty. Adults with intellectual disabilities had the highest proportion of Non-Hispanic Black/African American individuals (21.32% vs 11.67% in the full sample). Educational attainment varied significantly: 14.9% of adults with no functional limitation had less than a high school degree, compared to 26.24% with developmental disabilities and 46.39% with intellectual disabilities. Only 10% of adults with intellectual disabilities completed more than high school, compared to over 60% of adults with no functional limitations.

Employment disparities were notable, with 79.04% of adults with intellectual disabilities and 67.91% with developmental disabilities not in the labor force, compared to 22.19% of those with no functional limitations. Poverty rates were higher among adults with intellectual (25.32%) and developmental disabilities (20.44%) than those with no functional limitation (9.21%).

Insurance type also differed: 65.4% of adults with no functional limitation used private insurance, whereas adults with I/DD primarily relied on Medicaid (46.0% for intellectual disabilities, 29.63% for developmental disabilities) and Medicare (34.94% for intellectual disabilities, 32.66% for developmental disabilities).

These demographic differences highlight higher poverty and unemployment rates and lower educational attainment among adults with I/DD. χ^2^ tests confirmed statistically significant relationships between disability status and all demographic variables, indicating that disability status is linked to race, sex, education, employment, poverty, region, and healthcare coverage.

### Health Status

Logistic regression models (Appendix Table S1a) suggest adults with intellectual or developmental disabilities consistently had significantly higher odds of reporting poor or fair health compared to those with no functional limitations (*P* < .001) (Supplementary materials in the online appendix). In 2014-2018, adults with developmental disabilities had over 8 times greater odds of reporting poor or fair health (adjusted odds ratio [aOR] = 8.6, *P* < .001). Adults with intellectual disabilities had 11.59 times greater odds in 2009-2013 and 7.24 times greater odds in 2014-2018 of reporting poor or fair health compared to those with no functional limitations(*P* < .001).

### Healthcare access, affordability, and utilization

Appendix Table S1b-d feature logistic regression models for healthcare access, affordability, and utilization (Supplementary materials in the online appendix). Adults with intellectual or developmental disabilities were less likely to be uninsured or lack a usual source of care compared to those with no functional limitations. However, they had higher odds of forgoing or delaying medical care due to cost. Adults with I/DD were also more likely to have seen or talked to a health professional in the past 12 months compared to those without functional limitations.

### Trends in health Status and healthcare access, affordability, and utilization

**Figure 1.**
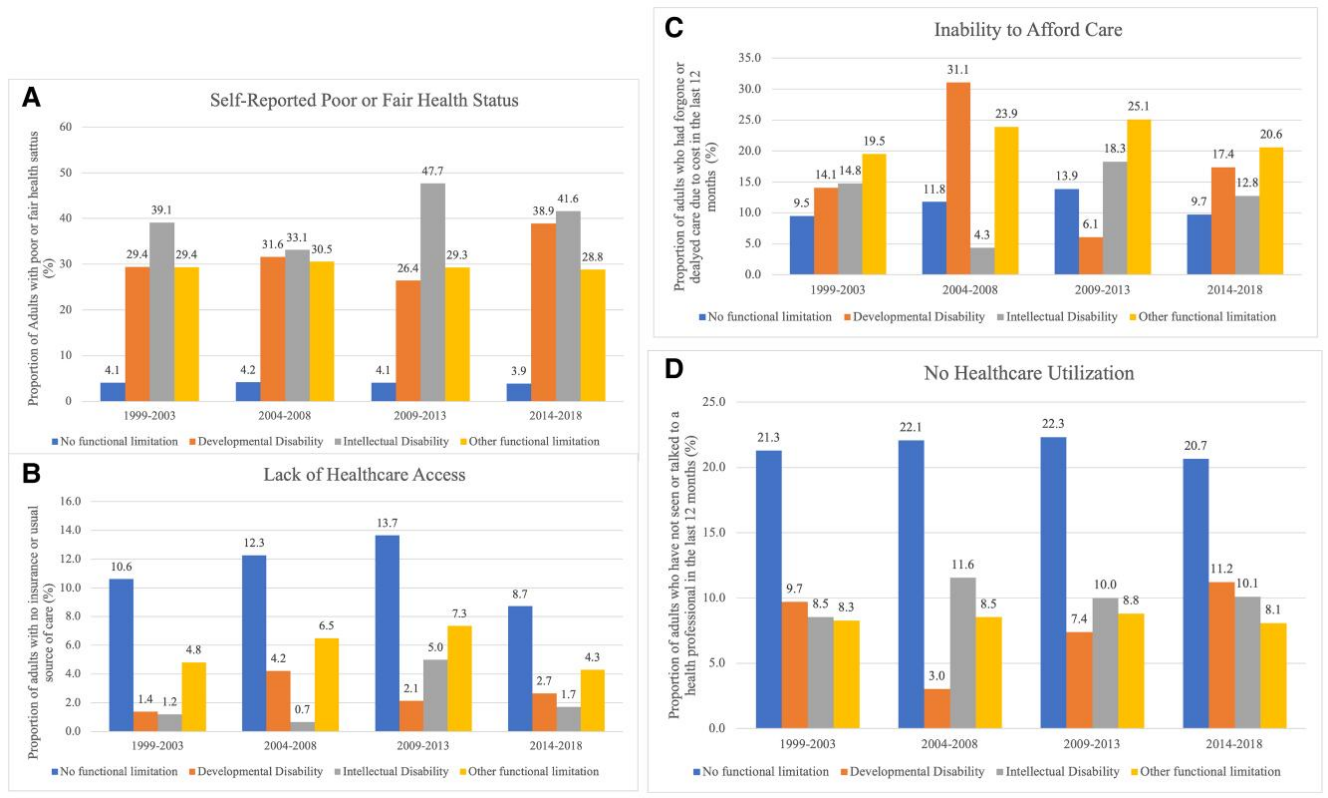
Health and healthcare outcomes (A-D) by disability status, National Health Interview Survey 1999-2018.

Figure 1A–D illustrates trends in health and healthcare outcomes. Adults without functional limitations showed consistent outcomes, with <5% reporting poor or fair health over 20 years. Adults with other functional limitations reported poor or fair health at 27%-30%, while those with intellectual disabilities reported 33%-47%, and those with developmental disabilities reported 26%-38%. The proportion of adults unable to afford care fluctuated most. For example, adults with developmental disabilities who could not afford care rose to 31% in 2004-2008, then fell to 6% in 2009-2013.

Table 2 estimates increased odds of forgoing or delaying care due to cost from 2005-2018 compared to 1999-2003 for both those with (*P* < .001) and without functional limitations (*P* < .001). There was also an increased risk of being uninsured and having no usual source of care from 2005 to 2018 for those without functional limitations (*P* < .001) and for those with functional limitations from 2005 to 2013. No significant changes were observed for adults with I/DD from 2005-2018 compared to 1999-2003.

**Table 2.** Changes in health status and healthcare access, affordability, and utilization for adults with and without I/DD, NHIS 1999-2018.

	Health status (poor or fair health status)	Healthcare affordability (forgone or delayed care due to cost)	Healthcare utilization (not seen/talked to health professional in past 12 months)	Healthcare access (no health insurance or usual source of care)
	aOR (95% CI)	aOR (95% CI)	aOR (95% CI)	aOR (95% CI)
No functional limitation				
Year (ref:1999-2003)				
2005-2008 (*N* = 86 895)	1.05 [0.99-1.1]^b^	1.28 [1.2-1.3]^a^	1.05 [1.0-1.1]^b^	1.19 [1.1-1.3]^a^
2009-2013 (*N* = 76 465)	1.02 [0.96-1.1]	1.55 [1.5-1.6]^a^	1.11 [1.1-1.14]^a^	1.44 [1.4-1.5]^a^
2014-2018 (*N* = 114 102)	1.02 [0.96-1.1]	1.06 [1.0-1.1]^b^	1.01 [0.98-1.0]	0.84 [0.79-0.88]^a^
Developmental disability				
Year (ref:1999-2003)				
2005-2008 (*N* = 112)	1.15 [0.57-2.3]	2.41 [1.1-5.1]^b^	0.31 [0.09-1.0]^b^	2.29 [0.40-13]
2009-2013 (*N* = 124)	1.03 [0.49-2.1]^b^	0.42 [0.17-0.99]^b^	0.83 [0.56-2.7]	1.13 [0.19-6.7]
2014-2018 (*N* = 216)	1.55 [0.83-2.9]	1.31 [0.66-2.6]	1.48 [0.56-3.9]	1.62 [0.30-8.7]
Intellectual disability				
Year (ref:1999-2003)				
2005-2008 (*N* = 128)	0.83 [0.45-1.6]	0.29 [0.09-0.87]^b^	1.36 [0.45-4.0]	0.56 [0.07-4.7]
2009-2013 (*N* = 198)	1.71 [0.95-3.1]^b^	1.55 [0.72-3.3]	0.98 [0.35-2.7]	5.06 [0.91-28.25]^b^
2014-2018 (*N* = 338)	1.06 [0.61-1.9]	0.99 [0.49-1.9]	1.01 [0.38-2.6]	1.15 [0.17-7.9]
Other functional limitation				
Year (ref:1999-2003)				
2005-2008 (*N* = 44 440)	1.07 [1.0-1.1]^a^	1.34 [1.3-1.4]^a^	1.05 [0.99-1.1]	1.43 [1.3-1.5]^a^
2009-2013 (*N* = 45 339)	1.01 [0.97-1.1]	1.42 [1.4-1.5]^a^	1.11 [1.0-1.2]^a^	1.67 [1.5-1.8]^a^
2014-2018 (*N* = 75 262)	1.03 [0.99-1.1]^b^	1.17 [1.1-1.2]^a^	1.07 [1.0-1.1]^b^	1.03 [0.94-1.1]

This table presents the adjusted odds ratios from multivariate logistic regression predicting changes in health status and healthcare access, affordability, and utilization for sample adults (18+) with and without I/DD, 1999-2018. aOR = adjusted odds ratio, adjusting for race, ethnicity, sex, age, poverty status, educational attainment, employment, and region of residence. Source: Author's analysis of data from the National Health Interview Survey, 1999-2018.

Abbreviations: I/DD, intellectual and developmental disabilities; NHIS, National Health Interview Survey.

^a^ *P* < .001; ^b^*P* < .151.

Post-hoc logistic regression models suggested adults with developmental disabilities (aOR = 7.62), intellectual disabilities (aOR = 8.71), and other functional limitations (aOR = 7.14) had significantly higher odds of reporting poor or fair health (*P* < .001). Adults with developmental disabilities had lower odds of forgoing or delaying care due to cost in 2008-2013 compared to 1999-2003. Other interaction terms were not statistically significant.

## Discussion

From 1999 to 2018, health disparities persisted for adults with I/DD in the United States. Despite policy changes and advancements in medicine and technology, significant health status differences between adults with and without I/DD remain.

The findings demonstrate that adults with I/DD are actively utilizing the healthcare system; however, challenges such as financial barriers to medical care persist, contributing to ongoing health disparities. This points to the possibility that enhancing health outcomes may require approaches beyond conventional healthcare services. For instance, increasing provider reimbursement rates could improve access, in addition to addressing the affordability of specialized medical equipment, diagnostic tests, and prescription medications is also essential for better health outcomes.

This study corroborates previous research highlighting sociodemographic disparities among adults with I/DD, who exhibit lower educational attainment and higher rates of poverty and unemployment.^32-34^ A significant proportion of this population relies on Medicaid and Medicare, which have been essential in increasing access to necessary healthcare services.^35^ While these programs are vital for reducing out-of-pocket costs compared to private health insurance, it is important to recognize that eligibility for these programs may arise from having an intellectual or developmental disability. Consequently, the challenges faced by this population may be influenced by broader systemic issues, including racism and ableism, which contribute to the ongoing health disparities we observe.^5^

Health status for adults with I/DD did not improve, remaining significantly worse than for those without disabilities. Despite greater access and utilization of healthcare, health outcomes did not reflect significant improvement. Notably, following the full implementation of the ACA in 2014, adults with no functional limitations, other functional limitations, and developmental disabilities experienced increases in their ability to afford care; however, adults with developmental disabilities also saw a rise in the rates of unaffordability for care compared to the period of 2009-2013. However, overall health status did not improve alongside gains in any affordability throughout the study period. Research will be needed to parse out this relationship.

These findings suggest that improvements in access, affordability, and utilization alone are insufficient to enhance health outcomes for adults with I/DD.^18,19^ Future policies and public health initiatives should prioritize not only the quality of care provided through Medicaid and Medicare but also address factors outside of traditional healthcare that impact health status. This includes enhancing access to social programs, promoting employment opportunities, and identifying pathways to supplement income for adults with I/DD. Moreover, healthcare providers could play a broader role by collaborating with vocational rehabilitation services and the employment sector to create more work opportunities for this population. Research should further explore the types of care most utilized by adults with I/DD, given their lower rates of primary, preventive, and specialized care visits and higher rates of emergency department use. Additionally, efforts to improve care quality should include adequate provider preparation, inclusive preventive practices, higher Medicaid reimbursement rates, and equitable public health initiatives.

### Limitations

This study has several limitations. Outcomes were based on self-reported data and did not include any triangulating measures of health status, and healthcare access, utilization, and affordability, which may introduce bias. However, these outcomes have been used extensively in prior research.^22^ Nonresponse could bias the results, though the NHIS has several strategies for mitigating this. Changes in diagnostic language and the stigmatization of disabilities may have also resulted in fewer self-reports of I/DD. The cross-sectional nature of this study limits our ability to assess temporal changes in health outcomes and healthcare access, as it captures a snapshot of data at a single point in time rather than longitudinal changes or trends over history.

Moreover, this study exclusively examines a non-institutionalized sample of individuals with I/DD, which may not fully represent the broader population, potentially overstating or understating the findings. Additionally, survey respondents needed the capacity to answer NHIS questions, potentially excluding non- or minimally speaking individuals with I/DD, thus affecting the sample's representativeness. We acknowledge that some responses may have been provided by proxies, though details into the extent of proxy reporting within the sample are unknown.

## Conclusion

Despite targeted policies and increased spending, health disparities for adults with I/DD have persisted with little progress. While access, affordability, and utilization are critical, they are insufficient to enhance health outcomes without addressing systemic barriers and gaps in care quality. Future research should investigate the healthcare experiences of adults with I/DD to identify areas for improvement, while also focusing on the causal impact of Medicaid policies on health, educational achievement, economic participation, and community integration. Current healthcare policies in the United States must prioritize accessibility and quality of care beyond just improving affordability and utilization.

## Supplementary Material

qxae158_Supplementary_Data

## References

[1] Zablotsky B, Black LI, Maenner MJ, et al Prevalence and trends of developmental disabilities among children in the United States: 2009-2017. Pediatrics. 2019;144(4):e20190811. 10.1542/peds.2019-0811 31558576 PMC7076808

[2] Hodges H, Fealko C, Soares N. Autism spectrum disorder: definition, epidemiology, causes, and clinical evaluation. Transl Pediatr. 2020;9(S1):S55–S65. 10.21037/tp.2019.09.09 32206584 PMC7082249

[3] Scheepers M, Kerr M, O’Hara D, et al Reducing health disparity in people with intellectual disabilities: a report from health issues special interest research group of the international association for the scientific study of intellectual disabilities. J Policy Pract Intellect Disabil. 2005;2(3-4):249–255. 10.1111/j.1741-1130.2005.00037.x

[4] McLean KJ, Bishop L. Chronic health conditions among adults with intellectual and developmental disabilities in a state Medicaid system. Am J Intellect Dev Disabil. 2024;129(5):331–345. 10.1352/1944-7558-129.5.331 39197849

[5] Magaña S, Parish S, Morales MA, Li H, Fujiura G. Racial and ethnic health disparities among people with intellectual and developmental disabilities. Intellect Dev Disabil. 2016;54(3):161–172. 10.1352/1934-9556-54.3.161 27268472

[6] Croen LA, Zerbo O, Qian Y, et al The health status of adults on the autism spectrum. Autism. 2015;19(7):814–823. 10.1177/1362361315577517 25911091

[7] Bishop-Fitzpatrick L, Rubenstein E. The physical and mental health of middle aged and older adults on the autism spectrum and the impact of intellectual disability. Res Autism Spectr Disord. 2019;63:34–41. 10.1016/j.rasd.2019.01.001 31768189 PMC6876625

[8] Salvador-Carulla L, Reed GM, Vaez-Azizi LM, et al Intellectual developmental disorders: towards a new name, definition and framework for “mental retardation/intellectual disability” in ICD-11. World Psychiatry. 2011;10(3):175–180. 10.1002/j.2051-5545.2011.tb00045.x 21991267 PMC3188762

[9] Anderson LL, Humphries K, McDermott S, Marks B, Sisirak J, Larson S. The state of the science of health and wellness for adults with intellectual and developmental disabilities. Intellect Dev Disabil. 2013;51(5):385–398. 10.1352/1934-9556-51.5.385 24303825 PMC4677669

[10] Bishop-Fitzpatrick L, Kind AJH. A scoping review of health disparities in autism spectrum disorder. J Autism Dev Disord. 2017;47(11):3380–3391. 10.1007/s10803-017-3251-9 28756549 PMC5693721

[11] Havercamp SM, Scandlin D, Roth M. Health disparities among adults with developmental disabilities, adults with other disabilities, and adults not reporting disability in North Carolina. Public Health Rep. 2004;119(4):418–426. 10.1016/j.phr.2004.05.006 15219799 PMC1497651

[12] Krahn GL, Hammond L, Turner A. A cascade of disparities: health and health care access for people with intellectual disabilities. Ment Retard Dev Disabil Res Rev. 2006;12(1):70–82. 10.1002/mrdd.20098 16435327

[13] Havercamp SM, Scott HM. National health surveillance of adults with disabilities, adults with intellectual and developmental disabilities, and adults with no disabilities. Disabil Health J. 2015;8(2):165–172. 10.1016/j.dhjo.2014.11.002 25595297

[14] Horner-Johnson W, Dobbertin K, Lee JC, Andresen EM, Expert Panel on Disability and Health Disparities. Disparities in health care access and receipt of preventive services by disability type: analysis of the medical expenditure panel survey. Health Serv Res. 2014;49(6):1980–1999. 10.1111/1475-6773.12195 24962662 PMC4254135

[15] Williamson HJ, Contreras GM, Rodriguez ES, Smith JM, Perkins EA. Health care access for adults with intellectual and developmental disabilities: a scoping review. OTJR (Thorofare N J). 2017;37(4):227–236. 10.1177/1539449217714148 28703641

[16] Tyler CV, Schramm SC, Karafa M, Tang AS, Jain AK. Chronic disease risks in young adults with autism spectrum disorder: forewarned is forearmed. Am J Intellect Dev Disabil. 2011;116(5):371–380. 10.1352/1944-7558-116.5.371 21905805

[17] Holder M, Waldman HB, Hood H. Preparing health professionals to provide care to individuals with disabilities. Int J Oral Sci. 2009;1(2):66–71. doi:0.4248/ijos.09022 20687298 10.4248/ijos.09022 PMC3735794

[18] Minihan PM, Bradshaw YS, Long LM, et al Teaching about disability: involving patients with disabilities as medical educators. Disabil Stud Q. 2004;24(4). 10.18061/dsq.v24i4.883

[19] Kaye HS . Disability-related disparities in access to health care before (2008-2010) and after (2015-2017) the Affordable Care Act. Am J Public Health. 2019;109(7):1015–1021. 10.2105/AJPH.2019.305056 31095413 PMC6603452

[20] Kennedy J, Wood EG, Frieden L. Disparities in insurance coverage, health services use, and access following implementation of the Affordable Care Act: a comparison of disabled and nondisabled working-age adults. Inquiry. 2017;54:0046958017734031. 10.1177/0046958017734031 29166812 PMC5798675

[21] Gréaux M, Moro MF, Kamenov K, Russell AM, Barrett D, Cieza A. Health equity for persons with disabilities: a global scoping review on barriers and interventions in healthcare services. Int J Equity Health. 2023;22(1):236. 10.1186/s12939-023-02035-w 37957602 PMC10644565

[22] Rosenbaum S . The patient protection and affordable care act: implications for public health policy and practice. Public Health Rep. 2011;126(1):130. 10.1177/003335491112600118 21337939 PMC3001814

[23] Schieve LA, Clayton HB, Durkin MS, et al Comparison of perinatal risk factors associated with autism Spectrum disorder (ASD), intellectual disability (ID), and co-occurring ASD and ID. J Autism Dev Disord. 2015;45(8):2361–2372. 10.1007/s10803-015-2402-0 25739693 PMC4608497

[24] National Center for Health Statistics. National Health Interview Survey, 1999-2018. Public-use data file and documentation. Accessed February 2, 2023. https://www.cdc.gov/nchs/nhis/documentation/?CDC_AAref_Val=https://www.cdc.gov/nchs/nhis/data-questionnaires-documentation.htm

[25] Blewett LA, Rivera Drew JA, King ML, et al IPUMS Health Surveys: National Health Interview Survey, Version 7.4 [dataset]. IPUMS; 2024. 10.18128/D070.V7.4

[26] Blewett LA, Rivera Drew JA, King ML, Williams KCW. IPUMS Health Surveys: National Health Interview Survey, Version 6.4 [dataset]. IPUMS; 2019. 10.18128/D070.V6.4

[27] Centers for Disease Control and Prevention (CDC) . About the National Health Interview Survey. National Center for Health Statistics. March 3, 2022. Accessed March 25, 2022. https://www.cdc.gov/nchs/nhis/about/index.html

[28] Parsons VL, Moriarity C, Jonas K, Moore TF, Davis KE, Tompkins L. Design and Estimation for the National Health Interview Survey, 2006-2015. U.S. Department of Health and Human Services, Centers for Disease Control and Prevention, National Center for Health Statistics; 2014. Accessed October 16, 2024. https://pubmed-ncbi-nlm-nih-gov.ezproxy.library.wisc.edu/24775908/

[29] Mahajan S, Caraballo C, Lu Y, et al Trends in differences in health status and health care access and affordability by race and ethnicity in the United States, 1999-2018. JAMA. 2021;326(7):637. 10.1001/jama.2021.9907 34402830 PMC8371573

[30] Kreuter F, Valliant R. A survey on survey statistics: what is done and can be done in Stata. Stata J. 2007;7(1):1–21. 10.1177/1536867X0700700101

[31] Lee S, Lee DK. What is the proper way to apply the multiple comparison test? Korean J Anesthesiol. 2018;71(5):353. 10.4097/kja.d.18.00242 30157585 PMC6193594

[32] Lee CE, Taylor JL. A review of the benefits and barriers to postsecondary education for students with intellectual and developmental disabilities. J Spec Educ. 2022;55(4):234–245. 10.1177/00224669211013354

[33] Nord D, Hamre K, Nye-Lengerman K. Understanding Community Poverty, Housing, and Disability. Institute on Community Integration; 2014. Accessed October 16, 2024. https://rtc.umn.edu/prb/243/

[34] National Association of State Directors of Developmental Disabilities Services (NASDDDS). National Report 2022-23: Employment. NCI-IDD; 2024. Accessed October 16, 2024. https://www.google.com/search?q=National+Report+2022-23%3A+Employment.+NCI-IDD&oq=National+Report+2022-23%3A+Employment.+NCIIDD&gs_lcrp=EgZjaHJvbWUqBggAEEUYOzIGCAAQRRg7MgYIARBFGDsyBggCEEUYQNIBBzM5M2owajSoAgCwAgA&sourceid=chrome&ie=UTF-8#:~:text=IPS%2022%2D23%20National%20Report%20Chapter%202,https%3A//idd.nationalcoreindicators.org%20%E2%80%BA%202024/06

[35] Medicaid and CHIP Payment and Access Commission (MACPAC). Access in Brief: Adults with Intellectual Disabilities and Developmental Disabilities; 2024. Accessed October 15, 2024. https://www.macpac.gov/publication/access-in-brief-adults-with-intellectual-disabilities-and-developmental-disabilities-2-2/

