# Supplementary material for: Health disparities persist for adults with developmental disabilities: NHIS insights, 1999-2018
Source: Health Aff Sch. 2025 Apr 22;3(4):qxae158. doi: 10.1093/haschl/qxae158 (PMC12013709; doi:10.1093/haschl/qxae158)
Supplement: qxae158_Supplementary_Data [file qxae158_supplementary_data.zip › HealthAffairsSupplement_revision_clean.docx]

**Table 1a**

*Adjusted Odds of Self-Reported Poor or Fair Health Status by Demographic Covariates*

|  | **1999-2003** | **2004-2008** | **2009-2013** | **2014-2018** |
| --- | --- | --- | --- | --- |
| Disability (ref: no functional limitation) |  |  |  |  |
| Developmental Disability | 6.83 (4.3-10.8)* | 6.73 (3.9-11.6)* | 4.33 (2.7-7.0)* | 8.6 (5.9-12.7)* |
| Intellectual Disability | 6.99 (4.3-11.4)* | 5.53 (3.6-8.4)* | 11.59 (7.9-16.9)* | 7.24 (5.4-9.8)* |
| Other functional limitation | 6.72 (6.4-7.0)* | 6.78 (6.4-7.1)* | 6.99 (6.6-7.4)* | 7.19 (6.8-7.6)* |
| Age | 1.01 (1.01-1.013)* | 1.01 (1.010-1.013)* | 1.01 (1.006-1.009)* | 1.01 (1.004-1.007)* |
| Race (ref: Non-Hispanic White) |  |  |  |  |
| Non-Hispanic Black/African American | 1.79 (1.7-1.9)* | 1.74 (1.6-1.9)* | 1.71 (1.6-1.8)* | 1.55 (1.5-1.6)* |
| Non-Hispanic Asian or Pacific islander | 1.31 (1.1-1.5)** | 1.16 (1.0-1.3)** | 1.43 (1.3-1.6)* | 1.25 (1.1-1.4)* |
| Hispanic/Latino | 1.53 (1.4-1.6)* | 1.44 (1.3-1.5) | 1.42 (1.3-1.5)* | 1.38 (1.3-1.5)* |
| Non-Hispanic Other | 1.58 (1.3-1.9)* | 1.45 (1.2-1.8)** | 1.58 (1.3-1.9)* | 1.48 (1.3-1.7)* |
| Sex (ref: male) |  |  |  |  |
| Female | 0.77 (0.7-0.8)* | 0.81 (0.8-0.84)* | 0.80 (0.76-0.84)* | 0.77 (0.7-0.8)* |
| Education (ref: Bachelor's degree higher) |  |  |  |  |
| less than high school | 3.59 (3.4-3.8)* | 3.17 (2.9-3.4)* | 3.33 (3.1-3.6)* | 3.27 (3.1-3.5)* |
| high school diploma or GED | 2.25 (2.1-2.4)* | 2.10 (1.9-2.3)* | 2.37 (2.2-2.6)* | 2.09 (1.9-2.2)* |
| Some college | 1.75 (1.6-1.9)* | 1.69 (1.6-1.8)* | 1.75 (1.6-1.9)* | 1.79 (1.7-1.9)* |
| Employment Status (ref: employed) |  |  |  |  |
| Unemployed | 1.50(1.3-1.7)* | 1.29 (1.1-1.2)* | 1.45 (1.3-1.6)* | 1.51 (1.3-1.7)* |
| Not in the labor force | 2.56 (2.4-2.7)* | 2.50 (2.4-2.6)* | 2.66 (2.5-2.8)* | 2.50 (2.4-2.6)* |
| Poverty Status (ref: at above poverty threshold) |  |  |  |  |
| Below poverty threshold | 1.77 (1.7-1.9)* | 1.86 (1.7-1.9)* | 1.99 (1.9-2.1)* | 1.88 (1.8-1.9)* |
| Region (ref: Northeast) |  |  |  |  |
| North Central/Midwest | .99 (0.9-1.1) | 1.06 (.99-1.1)** | 0.97 (0.9-1.1) | 0.99 (0.9-1.1) |
| South | 1.37 (1.3-1.5)* | 1.37 (1.3-1.5)* | 1.24 (1.1-1.3)* | 1.16 (1.1-1.2)* |
| West | 1.09 (1.0-1.2)** | 1.14 (1.1-1.2)* | 1.03 (0.9-1.1) | 1.03 (0.9-1.1) |

*Note.*  This table presents the adjusted odds of self-reporting poor or fair health status by all demographic covariates, adjusting for race, ethnicity, sex, age, poverty status, educational attainment, employment, and region of residence. * indicates p < .001; **indicates p < .151

**Table 1b**

*Adjusted Odds of Uninsured Status and Lack of Usual Source of Care by Demographic Covariates*

|  | **1999-2003** | **2004-2008** | **2009-2013** | **2014-2018** |
| --- | --- | --- | --- | --- |
| Disability (ref: no functional limitation) |  |  |  |  |
| Developmental Disability | 0.10 (0.2-0.4)** | 0.22 (0.5-0.9)** | 0.07 (0.02-0.2)* | 0.19 (0.1-0.5)** |
| Intellectual Disability | 0.06 (0.01-0.4)** | 0.03 (0.006-0.1)* | 0.25 (0.1-0.7)** | 0.12 (0.03-0.3)* |
| Other functional limitation | 0.80 (0.7-0.9)* | 0.93 (0.9-1.0)** | 0.88 (0.8-0.9)* | 0.84 (0.8-0.9)* |
| Age | 0.95 (0.951-0.96)* | 0.95 (0.95-0.96)* | 0.96 (0.95-0.96)* | 0.96 (0.96-0.97)* |
| Race (ref: Non-Hispanic White) |  |  |  |  |
| Non-Hispanic Black/African American | 1.26 (1.2-1.4)* | 1.24 (1.1-1.3)* | 1.25 ()1.1-1.4)* | 1.14 (1.0-1.3)** |
| Non-Hispanic Asian or Pacific islander | 2.29 (1.9-2.7)* | 1.55 (1.3-1.8)* | 1.69 (1.5-1.9)* | 1.34 (1.2-1.5)* |
| Hispanic/Latino | 3.34 (3.1-3.6)* | 3.14 (2.9-3.3)* | 2.78 (2.6-2.9)* | 2.69 (2.5-2.9)* |
| Non-Hispanic Other | 1.42 (1.1-1.8)** | 1.38 (0.9-1.9)** | 2.78 (2.6-2.9)** | 1.18 (0.9-1.6) |
| Sex (ref: male) |  |  |  |  |
| Female | 0.49 (0.47-0.5)* | 0.55 (0.5-0.6)* | 0.54 (0.5-0.6)* | 0.57 (0.5-0.6)* |
| Education (ref: Bachelor's degree higher) |  |  |  |  |
| less than high school | 5.50 (5.0-6.0)* | 6.01 (5.4-6.7)* | 5.39 (4.9-6.0)* | 6.09 (5.4-6.8)* |
| high school diploma or GED | 3.15 (2.9-3.4)* | 3.68 (3.3-4.0)* | 3.78 (3.4-4.1)* | 3.63 (3.3-4.0)* |
| Some college | 1.87 (1.7-2.0)* | 1.97 (1.8-2.2)* | 2.18 (1.9-2.4)* | 2.06 (1.9-2.3)* |
| Employment Status (ref: employed) |  |  |  |  |
| Unemployed | 3.92 (3.4-4.5)* | 3.24 (2.8-3.7)* | 3.10 (2.8-3.4)* | 2.34 (2.1-2.6)* |
| Not in the labor force | 0.79 (0.7-0.9)* | 0.62 (0.6-0.7)* | 0.58 (0.5-0.6)* | 0.55 (0.5-0.6)* |
| Poverty Status (ref: at above poverty threshold) |  |  |  |  |
| Below poverty threshold | 2.34 (2.1-2.6)* | 2.26 (2.1-2.5)* | 2.25 (2.1-2.4)* | 2.28 (2.1-2.4)* |
| Region (ref: Northeast) |  |  |  |  |
| North Central/Midwest | 1.12 (0.9-1.2)** | 1.33 (1.2-1.5)* | 1.23 (1.1-1.4)** | 1.43 (1.2-1.7)* |
| South | 1.89 (1.7-2.1)* | 2.37 (2.1-2.6)* | 2.16 (1.9-2.4)* | 2.54 (2.2-2.9)* |
| West | 1.63 (1.5-1.8)* | 1.86 (1.6-2.1)* | 1.74 (1.6-1.9)* | 1.45 (1.3-1.7)* |

*Note.* This table presents the adjusted odds of self-reporting not having health insurance or a usual source of care, adjusting for race, ethnicity, sex, age, poverty status, educational attainment, employment, and region of residence. * indicates p < .001; **indicates p < .151

**Table 1c**

*Adjusted Odds of Forgone or Delayed Medical Care Due to Cost by Demographic Covariates*

|  | **1999-2003** | **2004-2008** | **2009-2013** | **2014-2018** |
| --- | --- | --- | --- | --- |
| Disability (ref: no functional limitation) |  |  |  |  |
| Developmental Disability | 1.44 (0.9-2.4) | 2.87 (1.6-5.1)* | 0.34 (0.2-0.6)** | 1.91 (1.2-2.9)** |
| Intellectual Disability | 1.13 (0.6-2.1) | 0.28 (0.1-0.7)** | 1.38 (0.9-2.2) | 1.22 (0.8-1.8) |
| Other functional limitation | 3.01 (2.9-3.1)* | 3.19 (3.1-3.3)* | 2.81 (2.7-2.9)* | 3.14 (3.0-3.3)* |
| Age | 0.98 (0.978-0.98)* | 0.97 (0.977-0.98)* | 0.98 (0.7-0.98)* | 0.98 (0.982-0.985)* |
| Race (ref: Non-Hispanic White) |  |  |  |  |
| Non-Hispanic Black/African American | 1.03 (0.9-1.1) | 1.07 (1.0-1.3)** | 1.12 (1.1-1.2)* | 1.11 (1.1-1.2)* |
| Non-Hispanic Asian or Pacific islander | 0.60 (0.5-0.7)* | 0.57 (0.5-0.7)* | 0.64 (0.6-0.7)* | 0.65 (0.6-0.7)* |
| Hispanic/Latino | 0.93 (0.9-0.98)** | 1.08 (1.0-1.1)** | 1.09 (1.0-1.2)** | 1.07 (1.0-1.1)** |
| Non-Hispanic Other | 1.27 (1.0-1.5)** | 1.03 (0.8-1.3) | 0.99 (0.8-1.2) | 1.11 (0.9-1.4) |
| Sex (ref: male) |  |  |  |  |
| Female | 1.27 (1.2-1.3)* | 1.25 (1.2-1.3)* | 1.21 (1.2-1.3)* | 1.23 (1.2-1.3)* |
| Education (ref: Bachelor's degree higher) |  |  |  |  |
| less than high school | 1.98 (1.9-2.1)* | 1.90 (1.8-2.0)* | 1.92 (1.8-2.1)* | 1.83 (1.7-1.9)* |
| high school diploma or GED | 1.57 (1.5-1.7)* | 1.61 (1.5-1.7)* | 1.66 (1.6-1.8)* | 1.51 (1.4-1.6)* |
| Some college | 1.47 (1.4-1.6)* | 1.62 (1.5-1.7)* | 1.67 (1.6-1.8)* | 1.57 (1.5-1.6)* |
| Employment Status (ref: employed) |  |  |  |  |
| Unemployed | 2.41 (2.2-2.7)* | 2.24 (2.0-2.5)* | 1.96 (1.8-2.1)* | 1.82 (1.7-1.9)* |
| Not in the labor force | 0.93 (0.9-1.0)** | 0.82 (0.8-0.9)* | 0.76 (0.7-0.8)* | 0.76 (0.7-0.8)* |
| Poverty Status (ref: at above poverty threshold) |  |  |  |  |
| Below poverty threshold | 1.84 (1.7-1.9)* | 1.71 (1.6-1.8)* | 1.72 (1.6-1.8)* | 1.65 (1.6-1.7)* |
| Region (ref: Northeast) |  |  |  |  |
| North Central/Midwest | 1.04 (0.9-1.1) | 1.21 (1.1-1.3)* | 1.25 (1.2-1.3)* | 1.25 (1.2-1.3)* |
| South | 1.33 (1.2-1.4)* | 1.50 (1.4-1.6)* | 1.47 (1.4-1.6)* | 1.44 (1.4-1.5)* |
| West | 1.34 (1.2-1.4)* | 1.36 (1.3-1.5)* | 1.43 (1.3-1.5)* | 1.27 (1.2-1.4)* |

*Note.* This table presents the adjusted odds of self-reported forgone or delayed medical care due to cost, adjusting for race, ethnicity, sex, age, poverty status, educational attainment, employment, and region of residence. * indicates p < .001; **indicates p < .151

|  | **1999-2003** | **2004-2008** | **2009-2013** | **2014-2018** |
| --- | --- | --- | --- | --- |
| Disability (ref: no functional limitation) |  |  |  |  |
| Developmental Disability | 0.42 (0.2-0.9)** | 0.12 (0.05-0.3)* | 0.22 (0.1-0.5)** | 0.48 (0.2-0.9)** |
| Intellectual Disability | 0.35 (0.2-0.7)** | 0.39 (0.2-0.9)** | 0.37 (0.2-0.7)** | 0.39 (0.2-0.6)* |
| Other functional limitation | 0.46 (0.4-0.5)* | 0.47 (0.4-0.5)* | 0.48 (0.4-0.5)* | 0.50 (0.48-0.5)* |
| Age | 0.98 (0.98-0.985)* | 0.98 (0.980-0.983)* | 0.98 (0.97-0.98)* | 0.98 (0.980-0.982)* |
| Race (ref: Non-Hispanic White) |  |  |  |  |
| Non-Hispanic Black/African American | 0.95 (0.9-1.0)** | 0.99 (0.9-1.1) | 0.98 (0.9-1.0) | 0.96 (0.9-1.0) |
| Non-Hispanic Asian or Pacific islander | 1.66 (1.5-1.8)* | 1.53 (1.4-1.7)* | 1.61 (1.5-1.7)* | 1.50 (1.4-1.6)* |
| Hispanic/Latino | 1.57 (1.5-1.7)* | 1.68 (1.6-1.8)* | 1.58 (1.5-1.7)* | 1.49 (1.4-1.6)* |
| Non-Hispanic Other | 1.17 (0.9-1.4)** | 1.01 (0.8-1.3) | 1.22 (1.0-1.5)** | 1.28 (1.1-1.5)** |
| Sex (ref: male) |  |  |  |  |
| Female | 0.39 (0.4-0.41)* | 0.42 (0.4-0.44)* | 0.43 (0.4-0.5)* | 0.48 (0.46-0.49)* |
| Education (ref: Bachelor's degree higher) |  |  |  |  |
| less than high school | 2.27 (2.1-2.4)* | 2.25 (2.1-2.4)* | 2.22 (2.1-2.4)* | 2.25 (2.1-2.4)* |
| high school diploma or GED | 1.71 (1.6-1.8)* | 1.83 (1.7-1.9)* | 1.90 (1.8-2.0)* | 1.78 (1.7-1.9)* |
| Some college | 1.31 (1.2-1.4)* | 1.29 (1.2-1.4)* | 1.34 (1.3-1.4)* | 1.31 (1.3-1.4)* |
| Employment Status (ref: employed) |  |  |  |  |
| Unemployed | 1.29 (1.2-1.4)* | 1.25 (1.1-1.4)* | 1.29 (1.2-1.4)* | 1.19 (1.1-1.3)* |
| Not in the labor force | 0.68 (0.6-0.7)* | 0.68 (0.6-0.7)* | 0.66 (0.6-0.7)* | 0.62 (0.6-0.7)* |
| Poverty Status (ref: at above poverty threshold) |  |  |  |  |
| Below poverty threshold | 1.34 (1.3-1.4)* | 1.31 (1.2-14)* | 1.40 (1.3-1.5)* | 1.36 (1.3-1.4)* |
| Region (ref: Northeast) |  |  |  |  |
| North Central/Midwest | 1.35 (1.3-1.4)* | 1.26 (1.2-1.4)* | 1.25 (1.2-1.4)* | 1.30 (1.2-1.4)* |
| South | 1.44 (1.4-1.5)* | 1.34 (1.3-1.4)* | 1.31 (1.2-1.4)* | 1.36 (1.3-1.5)* |
| West | 1.57 (1.5-1.7)* | 1.49 (1.4-1.6)* | 1.49 (1.4-1.6)* | 1.42 (1.3-1.5)* |

**Table 1d**

*Adjusted Odds of Not Seeing a Health Professional in the Past 12 Months by Demographic Covariates*

*Note*. This table presents adjusted odds ratios of not seeing or talking to a health professional in the past 12 months, adjusting for race, ethnicity, sex, age, poverty status, educational attainment, employment, and region of residence. * indicates p < .001; **indicates p < .151.
